# Prevalence and Factors Associated with Substance Use Among Patients with Tuberculosis in Uganda

**DOI:** 10.21203/rs.3.rs-5927600/v1

**Published:** 2025-01-31

**Authors:** Ronald Olum, Damalie Nakkonde, Gloria Nassanga, Sarah Zalwango, Juliet N Sekandi

**Affiliations:** Makerere University School of Public Health; Makerere University School of Public Health; Makerere University School of Public Health; Kampala City Council Authority; University of Georgia

**Keywords:** Tuberculosis, Substance Use, Alcohol Use, Smoking, Uganda

## Abstract

**Background:**

Substance use can negatively impact treatment adherence and health outcomes, thus exacerbating the burden of the disease. This study determined the prevalence and factors associated with substance use among patients with TB disease in Kampala, Uganda.

**Methods:**

This was a cross-sectional study of 144 patients with drug-susceptible TB enrolled from July 2020 to March 2021 across five health facilities in Kampala. Eligible participants were 18–65 years old, diagnosed with TB, and had initiated treatment for <= one month. Exclusions included drug-resistant TB, severe illness, or impairments affecting study participation. Data on socio-demographics, substance use, and clinical characteristics were collected using a semi-structured questionnaire. Self-reported substance use was the outcome of interest. Descriptive statistics and simple logistic regression analyses were performed for factors associated with substance use. Stata version 18.0 was used for analysis.

**Results:**

The participants had a median age of 34 years (IQR: 25.5 – 45.0); 50% were female and 31.9% were HIV infected. The prevalence of any substance use was 20.8% among TB patients. Alcohol use was the predominant substance (18.1%), followed by marijuana (2.8%) and tobacco (2.1%). Males were more likely than females to use any substances (COR: 2.38, 95% CI: 1.02 – 5.56, p=0.055), as were HIV-infected persons (COR: 3.20, 95% CI: 1.40 – 7.34, p=0.006), and those affiliated with the Catholic religion (COR: 3.50, 95% CI: 1.06 – 11.60, p=0.040).

**Conclusion:**

Our study found a relatively high level of substance use among persons with TB. TB-HIV co-infected persons should be particularly targeted with interventions to minimize the negative health effects of substance use.

## INTRODUCTION

Tuberculosis (TB) remains a global public health challenge, with the World Health Organization (WHO) reporting approximately 10.6 million new cases and 1.3 million deaths worldwide in 2022 ^1^. Harmful substance use, particularly alcohol and smoking, accounts for an estimated 1.4 million cases of TB ^1^. Substance use does not only increase the risk of TB infection but can also impact disease progression, treatment adherence, and treatment outcomes ^2, 3^. In Uganda, TB poses a significant health burden, with 94,000 estimated cases and 1,900 deaths in 2022. Uganda is also among the top 30 countries with a high HIV/TB burden ^1^. Nearly one-third of TB cases in the country are linked to HIV, with substance abuse, including alcohol and smoking, as the second and third leading risk factors, resulting in 11,000 and 3,000 cases, respectively ^1^.

The relationship between substance use and TB outcomes is influenced by various biological and social mechanisms ^2^. Alcohol and drug use can impair immune function, increase the susceptibility to TB infection, and worsen disease severity. A recent meta-analysis found that alcohol consumption leads to 22.02 incident cases and 2.35 deaths per 100,000 individuals with tuberculosis ^4^. In Brazil, substance use, including alcohol and drug use alone, was significantly associated with an increased risk of death or loss-to-follow-up among patients with TB ^5^. Moreover, substance use disorders can indirectly lead to poverty, social destabilization, reduced access to healthcare, poor adherence to TB treatment regimens, and reduced bioavailability of medications, among others ^2, 6, 7^. Recent reviews have also found an association between substance use and the development of drug-resistant TB ^8, 9^. This relationship is likely to be secondary to non-adherence. A study done in Uganda found an independent relationship between substance use and drug-resistant TB; however, it was not significant after adjusting for confounders ^10^.

A few studies have examined the relationship between social and demographic characteristics of patients with TB and substance use in Uganda. Local research is needed to inform the development of targeted interventions among patients with active TB. There is only one published trial in Uganda that found that financial incentives increased alcohol abstinence among persons with latent TB, but the intervention did not impact adherence to treatment with isoniazid ^11^. This study aimed to determine the prevalence of substance use among patients with TB in Uganda and to identify associated demographic and clinical factors.

## METHODS

### Study Participants

This study analyzed baseline data from a cohort of 144 patients with active TB enrolled in a randomized controlled trial from July 2020 to March 2021. The trial aimed to evaluate the effectiveness of video-observed therapy in monitoring treatment adherence among patients with TB, and the detailed protocol has been published elsewhere ^12^. Participants were recruited from TB treatment clinics in five health facilities across Kampala, Uganda, including Lubaga and Mulago hospitals, as well as Kitebi, Kawaala, and Kisenyi Health Center IVs.

Eligibility criteria for participation in the original trial included adults aged 18–65 years with a confirmed diagnosis of drug-susceptible TB, categorized either as new cases or retreatment cases, who were treated for no more than one month at the time of enrollment. All participants were required to have provided informed consent, to have been residents of Kampala for the 6-month treatment period to ensure follow-up feasibility, and to be proficient in either Luganda or English. The exclusion criteria for this study included individuals who have any form of drug-resistant TB, those who are too ill to participate for the entire duration of the study, and those who have self-reported cognitive, motor, visual, or hearing disabilities that may hinder the effective use of the assigned intervention.

### Study Procedures

Data collection was collected using semi-structured interviews conducted by trained research assistants at the onset of the study. These interviews were based on a comprehensive questionnaire initially developed through an extensive review of existing literature to ensure it included all relevant aspects of the study. The questionnaire was also translated into Luganda, the most widely spoken local language, and back-translated into English for accuracy.

### Measures

The primary outcome of this analysis was substance use, which refers to the consumption of at least one psychoactive substance, such as alcohol, tobacco, marijuana, shisha, pipe, or any other substance reported by the participants. The participants were asked to disclose any substances they use, and we also probed for the intensity of smoking (number of cigarettes per day) and alcohol consumption (number of drinks per sitting). The data collected also included sociodemographic and clinical information, such as age, sex, level of education, religion, marital status, employment status, household composition by age group, median household income, HIV status, previous TB treatment history, and willingness to disclose TB status to family.

### Statistical Analyses

Data analysis was performed in Stata 18 (StataCorp LLC, College Station, Texas, USA). Descriptive statistics described the participants’ baseline social and demographic characteristics. Continuous variables, like age and income, were described using medians and interquartile range (IQR). Categorical variables were presented as frequencies and percentages. The prevalence of substance use was calculated as the proportion of individuals reporting the use of alcohol, tobacco, and other substances. Cross-tabulations were conducted to explore the relationship between substance use and participants’ sociodemographics with a chi-square test. Univariate logistic regression analyses were performed to estimate the crude odds ratios (COR) of substance use associated with social and demographic characteristics. Given the modest number of substance use events (n = 30), multivariable analysis was not performed to avoid model overfitting. As most of the substance use was attributed to alcohol, we conducted additional subgroup analysis for alcohol use. All tests were two-tailed, and a p-value of less than 0.05 was considered to indicate statistical significance.

## RESULTS

**Figure 1:**
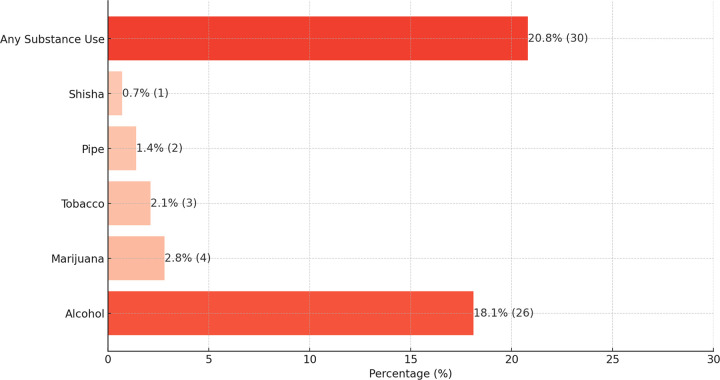
Types of substances used among patients on treatment for tuberculosis in Uganda.

A total of 144 patients with active tuberculosis were enrolled, with the gender split evenly between males and females (50% each). The median age was 34.0 years (interquartile range: 25.5–45.0), and nearly one-third (35.4%, n = 51) subscribed to Roman Catholicism. About 31.9% (n = 46) were living with HIV/AIDS, and 27.1% (n = 39) had a prior history of treatment for tuberculosis. Table 1 summarizes the social and demographic characteristics. The prevalence of substance use (Fig. 1) was 20.8% (n = 30), with alcohol (18.1%, n = 26) being the most used, followed by marijuana (2.8%, n = 4) and tobacco (2.1%, n = 3). Substance use significantly differed by gender (p = 0.040), religion (p = 0.009), and HIV status (p = 0.005), Table 2.

At simple logistic regression analysis (Table 2), male patients were more than twice as likely to use at least one substance than their female counterparts (crude odds ratio: 2.38, 95% CI: 1.02–5.56, p = 0.055). Participants living with HIV were thrice more likely to use substances than those without HIV (COR: 3.20, 95% CI: 1.40–7.34, p = 0.006). Compared to participants subscribing to Islam, Roman Catholics were 3.5 times more likely to use substances (COR: 3.50, 95% CI: 1.06–11.60, p = 0.040). On subgroup analysis (Table 3), male patients (COR: 2.63, 95% CI: 1.08–6.67, p = 0.034) and those living with HIV/AIDS (COR: 2.58, 95% CI: 1.08–6.13, p = 0.033) were more likely to use alcohol than their counterparts. Religion was not significantly associated with alcohol use.

## DISCUSSION

Our study aimed to determine the prevalence of substance abuse and the associated factors among patients with active TB disease who were receiving treatment in Kampala, Uganda. We found that 20.8% of patients with TB reported substance use, with alcohol being the most used. Substance use was significantly more prevalent among males, persons living with HIV/AIDS, and those affiliated with the catholic religion. These findings highlight the characteristics that could guide the development of targeted interventions for patients with TB who use substances.

The observed prevalence of substance use in our study is comparable to findings in Brazil, where substance among patients with TB was 22.2% ^5^. It is also similar to findings from a recent meta-analysis of smoking and alcohol use among patients with TB in Africa ^13^. In contrast, studies in South Africa reported a higher prevalence of smoking drugs (54.8%) among TB patients ^14^ and 32% harmful alcohol use, while 44% reported problematic drug use among hospitalized patients with TB ^15^. Similarly, a higher prevalence of substance use, ranging from 37.3–88%, was reported in Ethiopia ^16^, South Africa ^17, 18^, and Pakistan ^19^. In Uganda, substance use among patients with TB varies depending on the population studied. A previous reported that 17.5% of patients with TB were smokers, and 50.7% used alcohol ^20^. Among patients with DR-TB, the prevalence was 19% for smoking and 38.4% for using alcohol ^21^. Interestingly, the prevalence of substance use appears to be lower in non-African settings; for instance, in China, less than 10% of patients with TB reported smoking or alcohol use ^22^.

The variability in the prevalence of substance use among patients with TB across different settings can be attributed to several factors. First, cultural and social norms surrounding substance use differ significantly between countries, influencing both the consumption of substances and the likelihood of reporting such behavior ^23^. In countries like South Africa and Pakistan, where substance use among patients with TB was higher, these behaviors could be more socially acceptable within the population.

Second, differences in the assessment of substance use could impact the results. Most studies employed standard substance use tools such as the AUDIT, CAGE, MAST, and ASSIST. In contrast, our study used a single question to capture substance use, which could have led to an underestimation of the prevalence. Uganda has been cited as one of the highest consumers of alcohol in Africa, with an estimated annual per capita consumption of 12.2 liters ^24^. Alcohol consumption is widely accepted in some parts of Uganda ^25^, which should have contributed to a higher prevalence than observed in our study.

Our study identified significant differences in substance use prevalence based on sex, religious affiliation, and HIV status within the study population, which suggests underlying biological, social, and behavioral dynamics. The higher prevalence of substance use among males has been observed in other studies ^26, 27^. It is likely influenced by cultural and societal norms that are generally more permissive of substance use among men than women. In South Africa, substance use in male patients with TB is often driven by poverty ^28^. However, higher socioeconomic status can reduce substance use by increasing access to social support, which may positively impact treatment outcomes ^28^. This finding emphasized the need for gender-sensitive approaches in addressing substance use within TB care, ensuring that both prevention and treatment efforts are tailored to the specific challenges faced by male patients.

The association with religious affiliations may reflect varying cultural and religious norms surrounding substance use. For instance, Muslim religious teachings prohibit the consumption of alcohol, which may exert a strong protective effect against substance use. The association between HIV status and increased substance use among patients with TB points to a potential bidirectional relationship. On one hand, substance use can increase HIV risk by encouraging risky sexual behaviors. On the other hand, living with HIV may lead to greater substance as a coping mechanism for HIV-related stress ^29^. Therefore, there is a need for integrated care models that address both the medical and psychosocial needs of patients with both TB and HIV.

Our findings highlight the need for integrating screening for substance use and support into the standard TB care protocols. This holistic approach can improve adherence to TB treatment and mitigate the risk of unsuccessful treatment outcomes. Public health education on substance abuse should be culturally sensitive and tailored to the most vulnerable patients, such as those living with HIV/AIDS.

Our study had some limitations that must be considered when interpreting the findings. The cross-sectional design does not allow for causal inferences to be made. A limited sample of participants recruited from urban TB health facilities was included, limiting the findings’ generalizability. The measurement of substance use was based on one question, which limited the comparability with studies that used standardized tools. Future research that uses a longitudinal design, a larger sample, and standard substance abuse tools will yield more robust results.

### Conclusion

Our study found a relatively high level of substance use among persons with TB. Patients with both TB and HIV should be particularly targeted with interventions to minimize the negative health effects of substance use.

## Figures and Tables

**Table 1:** Social, demographic, and clinical characteristics of the study participants.

Characteristics (N = 144)	Frequency (Percentage)
**Age in complete years: median (IQR)**	34.0 (25.5–45.0)
**Age category in years**	
18–24	27 (18.8)
25–34	48 (33.3)
35–44	30 (20.8)
45–65	39 (27.1)
**Sex**	
Male	72 (50.0)
Female	72 (50.0)
**Highest level of education**	
No education or primary	55 (38.2)
Secondary	64 (44.4)
Tertiary/University	25 (17.4)
**Religion**	
Catholic	51 (35.4)
Protestant	33 (22.9)
Muslim	32 (22.2)
Other	28 (19.4)
**Marital Status**	
Married	55 (38.2)
Previously married	23 (16.0)
Single/never married	66 (45.8)
**Employment Status**	
Yes	90 (62.5)
No	54 (37.5)
**Household Age Groups**	
Less than 18 years old only	1 (0.7)
18 years old and above	45 (31.2)
Both less than 18 & above 18 years	88 (61.1)
**Household income (UGX): median (IQR)**	**200,000.0 (100,000.0–500,000.0)**
**HIV Status**	
Negative	98 (68.1)
Positive	46 (31.9)
**Previous TB Treatment**	
Yes	39 (27.1)
No	105 (72.9)
**Willingness to Disclose TB Status to Family**	
Yes	132 (91.7)
No	12 (8.3)

**Table 2:** Factors associated with substance use among patients with tuberculosis in Uganda.

Variables	Substance Use		Chi	Logistic Regression	
	No: n (%)	Yes: n (%)	P-value	Crude Odds ratio (95% CI)	P-value
**Age in complete years: median (IQR)**	**31.5 (20.0)**	**37.0 (15.0)**	**0.077**	**1.02 (0.99 to 1.06)**	**0.142**
**Age category**					
18–24	25 (21.9)	2 (6.7)	0.291	Ref	
25–34	37 (32.5)	11 (36.7)		3.72 (0.76 to 18.22)	0.106
35–44	23 (20.2)	7 (23.3)		3.80 (0.72 to 20.22)	0.117
45–65	29 (25.4)	10 (33.3)		4.31 (0.86 to 21.56)	0.075
**Sex**					
Female	62 (54.4)	10 (33.3)	0.040	Ref	
Male	52 (45.6)	20 (66.7)		2.38 (1.02–5.56)	0.044
**Highest level of education**					
No education or primary	41 (36.0)	14 (46.7)	0.195	Ref	
Secondary	55 (48.2)	9 (30.0)		0.48 (0.19 to 1.21)	0.121
Tertiary/University	18 (15.8)	7 (23.3)		1.14 (0.39 to 3.30)	0.811
**Religion**					
Muslim	28 (24.6)	4 (13.3)	0.009	Ref	
Catholic	34 (29.8)	17 (56.7)		3.50 (1.06 to 11.60)	0.040
Protestant	25 (21.9)	8 (26.7)		2.24 (0.60 to 8.35)	0.230
Other	27 (23.7)	1 (3.3)		0.26 (0.03 to 2.47)	0.241
**Marital Status**					
Married	41 (36.0)	14 (46.7)	0.492	Ref	
Previously married	18 (15.8)	5 (16.7)		0.81 (0.25 to 2.60)	0.728
Single/never married	55 (48.2)	11 (36.7)		0.59 (0.24 to 1.42)	0.237
**Employment Status**					
No	47 (41.2)	7 (23.3)	0.072	Ref	
Yes	67 (58.8)	23 (76.7)		2.30 (0.91 to 5.81)	0.077
**HIV Status**					
Negative	84 (73.7)	14 (46.7)	0.005	Ref	
Positive	30 (26.3)	16 (53.3)		3.20 (1.40 to 7.34)	0.006
**Previous TB Treatment**					
No	85 (74.6)	20 (66.7)	0.387	Ref	
Yes	29 (25.4)	10 (33.3)		1.47 (0.62 to 3.49)	0.388

**Table 3:** Factors associated with alcohol use among patients with tuberculosis in Uganda.

Variables	Alcohol Use		Chi	Logistic Regression	
	No	Yes	P-value	Crude Odds ratio (95% CI)	P-value
**Age in complete years**	31.5 (25.0–45.0)	37.0 (30.0–45.0)	0.126	1.02 (0.99 to 1.06)	0.246
**Age category**					
18–24	25 (21.2)	2 (7.7)	0.422	Ref	
25–34	39 (33.1)	9 (34.6)		2.88 (0.58 to 14.47)	0.198
35–44	23 (19.5)	7 (26.9)		3.80 (0.72 to 20.22)	0.117
45–65	31 (26.3)	8 (30.8)		3.23 (0.63 to 16.57)	0.161
**Sex**					
Female	64 (54.2)	8 (30.8)		Ref	
Male	54 (45.8)	18 (69.2)	0.030	2.58 (1.08–6.13)	0.034
**Highest level of education**					
No education or primary	45 (38.1)	10 (38.5)	0.307	Ref	
Secondary	55 (46.6)	9 (34.6)		0.74 (0.28 to 1.97)	0.542
Tertiary/University	18 (15.3)	7 (26.9)		1.75 (0.58 to 5.31)	0.323
**Religion**					
Muslim	28 (23.7)	4 (15.4)	0.060	Ref	
Catholic	38 (32.2)	13 (50.0)		2.39 (0.71 to 8.13)	0.161
Protestant	25 (21.2)	8 (30.8)		2.24 (0.60 to 8.35)	0.230
Other	27 (22.9)	1 (3.8)		0.26 (0.03 to 2.47)	0.241
**Marital Status**					
Married	43 (36.4)	12 (46.2)	0.606	Ref	
Previously married	20 (16.9)	3 (11.5)		0.54 (0.14 to 2.12)	0.375
Single/never married	55 (46.6)	11 (42.3)		0.72 (0.29 to 1.78)	0.473
**Employment Status**					
Yes	71 (60.2)	19 (73.1)	0.218	Ref	
No	47 (39.8)	7 (26.9)		1.80 (0.70 to 4.61)	0.223
**HIV Status**					
Negative	85 (72.0)	13 (50.0)	0.029	Ref	
Positive	33 (28.0)	13 (50.0)		2.58 (1.08 to 6.13)	0.033
**Previous TB Treatment**					
Yes	30 (25.4)	9 (34.6)	0.340	Ref	
No	88 (74.6)	17 (65.4)		1.55 (0.63 to 3.85)	0.342
